# Insecticide resistance in *Anopheles gambiae: *data from the first year of a multi-country study highlight the extent of the problem

**DOI:** 10.1186/1475-2875-8-299

**Published:** 2009-12-17

**Authors:** Hilary Ranson, Hiba Abdallah, Athanase Badolo, Wamdaogo Moussa Guelbeogo, Clément Kerah-Hinzoumbé, Elise Yangalbé-Kalnoné, N'Falé Sagnon, Frédéric Simard, Maureen Coetzee

**Affiliations:** 1 Vector Group, Liverpool School of Tropical Medicine, Pembroke Place, Liverpool, L3 5QA, UK; 2 Vector Biology & Control unit, Blue Nile National Institute for Communicable Disease, PO Box 101, Wad Medani, Sudan; 3 Centre National de Recherche et de Formation sur le Paludisme 01 BP 2208 Ouagadougou 01, Burkina Faso; 4 Université de Ouagadougou, BP 7021, Ouagadougou 03, Burkina Faso; 5 National Malaria Control Programme BP 1043, N'Djaména, Chad; 6 Institut de Recherche pour le Développement (IRD), UR016 and Institut de Recherche en Sciences de la Santé (IRSS), 01 BP 171 Bobo-Dioulasso, Burkina Faso; 7 Malaria Entomology Research Unit, School of Pathology, University of the Witwatersrand, Johannesburg 2000, South Africa; 8 Vector Control Reference Unit, National Institute for Communicable Diseases of the NHLS, Private Bag X4, Sandringham, Johannesburg 2131, South Africa

## Abstract

**Background:**

Insecticide resistance in malaria vectors is a growing concern in many countries which requires immediate attention because of the limited chemical arsenal available for vector control. The current extent and distribution of this resistance in many parts of the continent is unknown and yet such information is essential for the planning of effective malaria control interventions.

**Methods:**

In 2008, a network was established, with financial support from WHO/TDR, to investigate the extent of insecticide resistance in malaria vectors in five African countries. Here, the results of bioassays on *Anopheles gambiae sensu lato *from two rounds of monitoring from 12 sentinel sites in three of the partner countries are reported.

**Results:**

Resistance is very heterogeneous even over relatively small distances. Furthermore, in some sites, large differences in mortality rates were observed during the course of the malaria transmission season. Using WHO diagnostic doses, all populations from Burkina Faso and Chad and two of the four populations from Sudan were classified as resistant to permethrin and/or deltamethrin. Very high frequencies of DDT resistance were found in urban areas in Burkina Faso and Sudan and in a cotton-growing district in Chad. In areas where both *An. gambiae s.s*. and *Anopheles arabiensis *were present, resistance was found in both species, although generally at a higher frequency in *An gambiae s.s*. *Anopheles gambiae s.l*. remains largely susceptible to the organophosphate fenitrothion and the carbamate bendiocarb in the majority of the sentinel sites with the exception of two sites in Burkina Faso. In the cotton-growing region of Soumousso in Burkina Faso, the vector population is resistant to all four classes of insecticide available for malaria control.

**Conclusions:**

Possible factors influencing the frequency of resistant individuals observed in the sentinel sites are discussed. The results of this study highlight the importance of standardized longitudinal insecticide resistance monitoring and the urgent need for studies to monitor the impact of this resistance on malaria vector control activities.

## Background

The use of insecticides in malaria control programmes in Africa is expanding with the extensive and rapid roll out of long lasting insecticide-treated bed nets (LLINs) and indoor residual spraying (IRS) [1]. Twelve insecticides are approved by the World Health Organization (WHO) for IRS, but these belong to just four chemical classes (organochlorines, organophosphates, carbamates and pyrethroids) [2]. All four of these classes are nerve poisons and either target acetylcholinesterase in the synapses or the voltage-gated sodium channel on the insect neurones. For insecticide-impregnated material, such as LLINs, the chemical arsenal is even more limited with only six insecticides, all from the pyrethroid class, available [2]. These same insecticide classes are also widely used to control agricultural pests in Africa and this can pose additional selection pressure on mosquitoes when insecticide contaminated ground water permeates their larval habitats. This intensive exposure to insecticides has inevitably resulted in the evolution of insecticide resistance in the *Anopheles *mosquitoes that vector malaria. Resistance to the organochlorines DDT and the now obsolete dieldrin was first reported in African malaria vectors in the 1950s and 1960s [3,4]. Pyrethroid resistance was detected in African malaria vectors in 1993 [5]. Since then there have been published reports of pyrethroid resistant populations of *Anopheles gambiae s.l*. in countries from west, central, east and southern Africa (see, for example [6-9]) and *Anopheles funestus *in Ghana, Mozambique and South Africa [10,11]. Recently, carbamate and organophosphate resistant populations of *An. gambiae *have been reported in west Africa [12].

The WHO has produced a series of guidelines for measuring insecticide resistance in disease vectors and determined a set of 'diagnostic doses' for many of the commonly used insecticides [13]. A WHO reference centre in Malaysia supplies insecticide bioassay kits and papers coated with these diagnostic doses of insecticides. This has facilitated standardization of bioassays, but even when these guidelines are followed, variations in methodologies can make inter study comparisons difficult. For example, although WHO protocols specify using 3-5 day old unfed females, the difficulties in measuring mosquito age in the field and the lack of even basic insectary facilities needed to rear adult mosquitoes from field collected larvae, sometimes leads to the use of wild caught mosquitoes, which are of mixed age and physiological status [14]. This may confound results, as both age and blood feeding status are known to influence the mosquito's response to insecticides [15,16]. Difficulties in procuring the insecticide-treated papers from the WHO reference centre can also disrupt planned insecticide resistance monitoring and can sometimes necessitate the adoption of alternative methodologies, or the use of insecticide papers procured from alternative sources. In some cases molecular assays to detect the resistance alleles have been used as a substitute for bioassays [17]. These molecular assays have an important role to play in insecticide resistance management. As they work at the individual rather than population level and are able to identify heterozygotes that may have no discernable phenotype, they are more sensitive than bioassays. However, current understanding of the molecular basis of insecticide resistance in malaria vectors is largely limited to target site resistance. Given that this is only one of several mechanisms implicated in conferring resistance, the molecular tools cannot currently be used as a substitute for bioassays.

The WHO/TDR network on insecticide resistance in African malaria vectors was established in 2008. One of the major objectives of this network is to improve the monitoring of insecticide resistance in malaria vectors, prioritizing areas where large scale insecticide based control programmes are being implemented or planned in the absence of data on vector susceptibility. Here steps taken to standardize resistance monitoring in each of the participant countries are described and results from year one for three countries, Burkina Faso, Chad and Sudan, are reported. The bioassay data indicate an alarmingly high distribution of resistance in members of the *An. gambiae *complex. The implications for the sustainability of malaria vector control in Africa are discussed.

## Methods

### Description of study sites

Each country identified four sentinel sites for biannual resistance monitoring. These sites were chosen to encompass a range of insecticide selection pressures and included an urban site with local use of insecticides in subsistence agriculture, a rural site with intensive crop cultivation (cotton in the three countries reported in this study), a site with very high coverage of insecticide based malaria control interventions and a site with less insecticide usage (no intensive agriculture and no vertically organized vector control programmes). All sites were in malaria endemic regions and the boundaries were defined by a 10 km radius from the public health care facility.

Malaria transmission is seasonal in all three countries, and peaks at the end of the wet season. In Burkina Faso, the main rains last from June to October and in Chad, rainfall is highest from July to September. In Sudan the peak months of malaria transmission are from September to November, but in Gezira State there is an additional peak at the end of February, corresponding to the end of the irrigation season when small pools of water form along the drying canals and increase the mosquito density [18].

**Figure 1:**
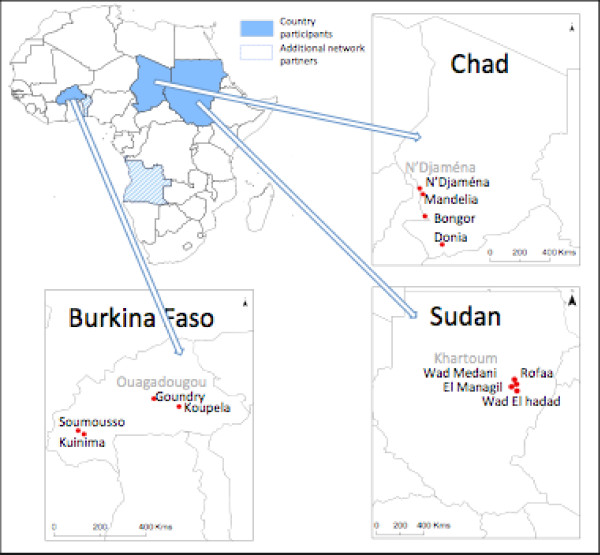
**Map showing the geographical locations of the study sites**. The WHO/TDR network has five member countries shown in blue on the map of Africa. Activities in Angola and Benin started midway through the 2008 malaria transmission season and a complete data set is therefore not available for these two countries. The sentinel sites described in the current study are shown as red circles on the individual country maps. Capital cities are shown in grey.

Two rounds of mosquito collections were performed in each of the 12 sites to coincide with the beginning and end of the peak malaria transmission season. The study sites are described in more detail below and their location is indicated in Figure 1.

#### Burkina Faso

The urban agriculture site is Kuinima (N 11° 09', W 04° 17'), which is located in the district of Bobo Dioulasso in the Sudan Savannah zone, south-west Burkina Faso. The local human population applies unknown quantities of insecticides to protect their vegetable crops from insect damage. Larval collections were made between 21st and 24^th ^August and 17^th ^and 20^th ^October 2008. Soumousso (N 11° 00', W 04° 03') is also located in the Sudan Savannah zone in the southwest of the country, but this village is in the heart of the cotton belt. Six rounds of insecticide (pyrethroids, organophosphates, and carbamates) are applied to the cotton crop between June and October each year. Larval collections were made between 18^th ^and 28^th ^August and 17^th ^and 20^th ^October 2008. Koupela, (N 12° 10', W 00° 21') is in the Sudan Sahelian zone approximately 130 km east of the capital city, Ouagadougou. Almost all households in Koupela received LLINs from the National Malaria Control Programme (NMCP) and other social partners in years 2004 to 2006. Larval collections were made between 30^th ^July and 27^th ^August and 28^th ^and 30^th ^October 2008. Finally, Goundry (N 12° 30', W 01° 20') 30 km from Ouagadougou has low levels of insecticide usage and has not yet been targeted by the NMCP for bed net distribution. Collection dates were 26^th ^July to 3^rd ^September and 29^th ^October to 30^th ^October 2008 for this site.

#### Chad

The urban agriculture site is in the capital city, N'djamena (N 12° 06', E 15° 02'). The average annual rainfall is 400 mm but the location of the site on the river Chari permits year-round vegetable cultivation by market gardeners and pyrethroid insecticides are regularly applied to protect the crops. Larval collections were made between 27^th ^July and 7^th ^August, and 8^th ^and 17^th ^September 2008. Donia, in the extreme south of Chad (N 8° 24', E 16° 24') has an annual rainfall of 1200 mm and is a site of intensive agricultural production. The main crops are cotton, vegetables and oil seeds. Here, the collection dates were 27^th ^August to 1^st ^September and 20^th ^to 22^nd ^October 2008. Bongor (N 10° 16', E 15° 21') is on the border with Cameroon. Rice is the main crop but insecticide usage is minimal on this crop. LLINs have been distributed to all children under one year and pregnant women. Larval collections in Bongor were made between 18^th ^and 23^rd ^August and 13^th ^and 19^th ^October 2008. The fourth site, Mandelia (N 11° 43', E 15° 14'), is located 50 km south of the capital in the Sudan Sahelian zone. Agricultural production is minimal at this site. The samples were collected between 15^th ^and 26^th ^July and 19^th ^and 29^th ^September 2008.

#### Sudan

The study sites in Sudan are in Gezira state, in the rich Savannah region of central Sudan. The urban agriculture site, Wad Medani (N 11° 24', E 33° 31') is in the Gezira agricultural irrigation scheme. The main crops are vegetables. Larval collections were made between 13^th ^and 14^th ^September 2008 and on 4^th ^January 2009. El Managil (N 14° 11', E 33° 08') is also located in the irrigation scheme, but is in a cotton growing area. Larval collections were made between 15^th ^and 16^th ^October 2008 and 16^th ^and 20^th ^January 2009. Wad El Hadad (N 13° 51', E 33° 33') is the vector control site. There is a very high coverage of LLINs in this site and the NMCP also uses temephos for larviciding and conducts IRS with bendiocarb. Larval collections were made between 26^th ^October and 3^rd ^November 2008 and 24^th ^and 27^th ^January 2009. Rofaa (N 14° 47', E 33° 22') is situated on the left bank of the Blue Nile outside the Gezira irrigation scheme. There is no known insecticide use for agricultural purposes in this site and the only vector control activity is larviciding with temephos. This was selected as the low insecticide pressure site and larval collections were made between 4^th ^and 8^th ^October 2008, and 10^th ^and 12^th ^January 2009.

### Insecticide bioassays

*Anopheles *larvae were collected from a range of larval habitats in each sentinel site and reared to adults in local insectaries. A minimum of 800 3^rd^-4^th ^instar larvae were collected from each study site during each collection round. Sampling was from multiple breeding sites within a 10 Km radius and performed over several days to minimize the probability of analyzing siblings. All samples were pooled and raised together to the adult stage.

*Anopheles gambiae s.l*. mosquitoes were morphologically identified and non-blood fed, 3-5 day old adult females were used for the insecticide bioassays. Insecticide papers were obtained from the WHO reference centre in Malaysia [19]. Each batch was tested on the insecticide susceptible Kisumu strain of *An gambiae *at the Liverpool School of Tropical Medicine before dispatch to the participant countries. Five insecticides were tested: 4% DDT, 0.75% permethrin, 0.05% deltamethrin, 0.1% bendiocarb and 1% fenitrothion. These insecticides belong to the organochlorine, type I pyrethroid, type II pyrethroid, carbamates and organophosphate classes respectively. Each paper was used a maximum of six times. A minimum of 100 mosquitoes (four replicates of approximately 25 mosquitoes, with tests performed over more than one day) were exposed to each insecticide for 1 hour. Mortality was recorded 24 hours after exposure. Control tests were run each day and Abbot's formula used to correct for control mortality where necessary. Data were recorded in an excel spreadsheet in a format ready for uploading into the insecticide resistance database (IRbase) currently under development [20]. WHO guidelines were used to classify populations as 'resistant' if less than 80 per cent mortality was observed and as 'suspected resistant' if mortality rates were between 80 and 98 per cent [13]. Surviving and dead mosquitoes were retained on silica gel for species identification.

### Identification of members of the *Anopheles gambiae *species complex

For each site, a random sample of 40 mosquitoes from the bioassay controls were identified using the PCR-RFLP technique described by Fanello *et al *[21] Species/molecular form identification was also performed on up to 40 survivors, (and in Burkina Faso on an equal number of dead mosquitoes) for each site, collection round and insecticide tested. The species distribution in the surviving and dead progeny in the Burkina Faso samples was compared to that in the total population used for the bioassays, weighted according to the species distribution in the population using the following formula: (((Number of species A in Survivors/Number of bioassay survivors identified by PCR)*total number of surviving progeny from bioassay)+((Number of species A in Dead/Number of bioassay dead identified by PCR)*total number or dead progeny from bioassay))/(Total number bioassayed). Species distribution in the bioassayed subset was compared to the distribution in the surviving subset, dead subset and control sample (exposed to control papers and used to estimate species distribution in each study site) using contingency table chi-squared statistics.

## Results

**Figure 2:**
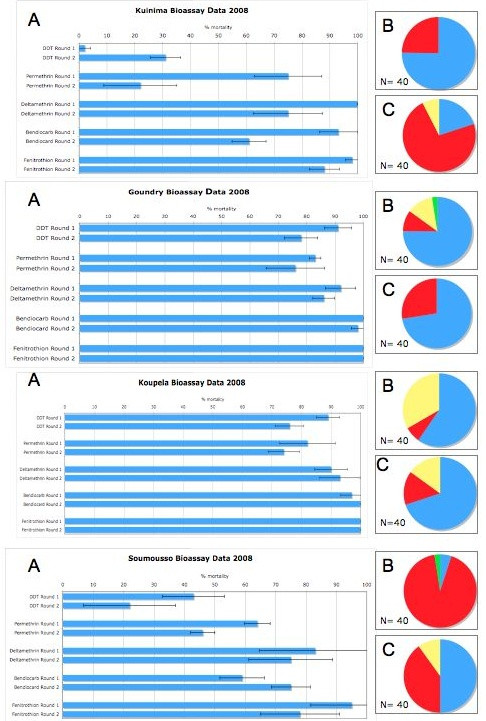
**Insecticide bioassay results for *Anopheles gambiae s.l*. from 2008/2009 in two rounds of monitoring in four sentinel sites in Burkina Faso**. Panel A shows percentage mortality 24 hours after a 1-hour exposure to the WHO diagnostic doses of insecticide. The minimum sample size for these assays was 100 and all individuals were non-blood fed females, 3-5 days post emergence. Panels B and C show the species distribution in each of the study sites during collection round one (Panel B) and round 2 (Panel C). The mosquitoes were morphologically identified as belonging to the *Anopheles gambiae *complex and then identified to species and molecular form by PCR. *Anopheles arabiensis *is shown in blue, *An. gambiae *S form in red, *An. gambiae *M form in yellow and *An. gambiae *M/S hybrids in green.

**Figure 3:**
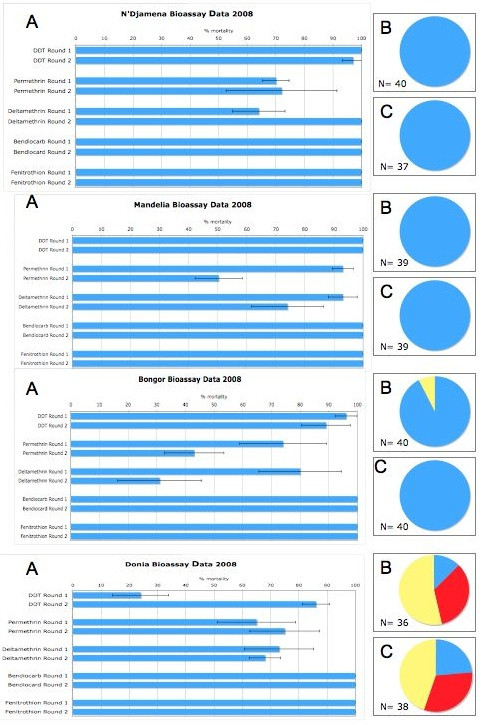
**Insecticide bioassay results for *Anopheles gambiae s.l*. from 2008/2009 in two rounds of monitoring in four sentinel sites in Chad**. Panel A shows percentage mortality 24 hours after a 1-hour exposure to the WHO diagnostic doses of insecticide. The minimum sample size for these assays was 100 and all individuals were non-blood fed females, 3-5 days post emergence. Panels B and C show the species distribution in each of the study sites during collection round one (Panel B) and round 2 (Panel C). The mosquitoes were morphologically identified as belonging to the *Anopheles gambiae *complex and then identified to species and molecular form by PCR. *Anopheles arabiensis *is shown in blue, *An. gambiae *S form in red, *An. gambiae *M form in yellow and *An. gambiae *M/S hybrids in green.

**Figure 4:**
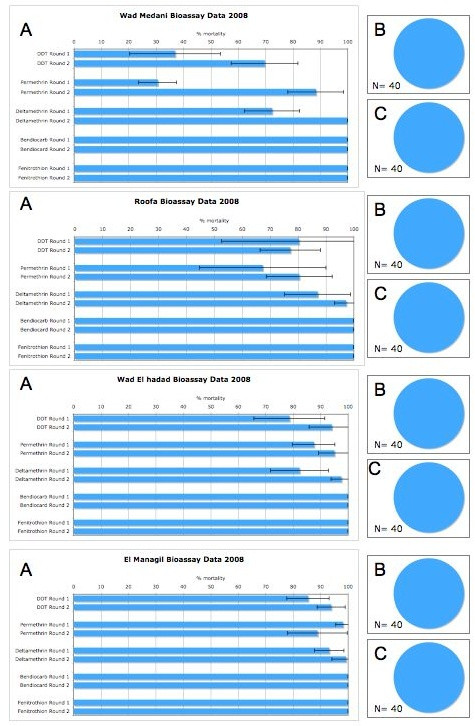
**Insecticide bioassay results for *Anopheles gambiae s.l*. from 2008/2009 in two rounds of monitoring in four sentinel sites in Sudan**. Panel A shows percentage mortality 24 hours after a 1-hour exposure to the WHO diagnostic doses of insecticide. The minimum sample size for these assays was 100 and all individuals were non-blood fed females, 3-5 days post emergence. Panels B and C show the species distribution in each of the study sites during collection round one (Panel B) and round 2 (Panel C). The mosquitoes were morphologically identified as belonging to the *Anopheles gambiae *complex and then identified to species and molecular form by PCR. *Anopheles arabiensis *is shown in blue, *An. gambiae *S form in red, *An. gambiae *M form in yellow and *An. gambiae *M/S hybrids in green.

The results of the insecticide bioassays are shown in Figures 2, 3 and 4. In Burkina Faso the highest percentage of mosquitoes surviving the WHO diagnostic doses were seen in the two agricultural districts in the southwest of the country, Soumousso and Kuinima. This agrees with earlier monitoring of resistance in this country completed in 2006 which found higher frequencies of resistance in the western part of the country than in the central and eastern parts [22]. However, the current study clearly demonstrates a general increase in the numbers of resistant *An. gambiae *in Burkina Faso during the past three years. Populations in the Ouagadougou district that were reported as fully susceptible to pyrethroids and DDT in 2006 [22] now show permethrin and DDT resistance, with mortality levels less than 80 per cent recorded in October 2008. The Soumousso population also shows a much lower mortality to DDT and pyrethroids than was reported previously. Resistance to bendiocarb and fenitrothion was also detected in Kuinima and Soumousso. This is the first published bioassay data on these insecticides in Burkina Faso although resistance has been inferred by the presence of the *ace-1*^*R *^mutation, in this region [23].

*Anopheles gambiae *M and S forms and *Anopheles arabiensis *were found in all four study sites in Burkina Faso and the relative frequencies of each form varied between the two collection seasons (Figure 2). The species distribution within the DDT and permethrin survivors and dead progeny was compared to that of the general population (weighted according to the bioassay results) for the second collection season (where resistance was generally higher) for all sites in Burkina Faso (Table 1). With two exceptions (permethrin survivors in Kuinima and DDT survivors in Soumousso), the species distribution in the surviving and dead subset differed significantly from the distribution in the total population used for the bioassays. In general, the majority of the DDT and permethrin survivors are comprised of the S form while the biggest proportion of the dead progeny was identified as *An. arabiensis*. It is important to note that, although lower mortality with the diagnostic dose was generally observed for *An. gambiae *compared to *An. arabiensis*, some individuals from the latter species survived the diagnostic doses of DDT and pyrethroids in almost all assays indicating that resistance is widely distributed, albeit currently at lower frequencies, in *An. arabiensis *in Burkina Faso.

**Table 1:** Species distribution in alive and dead progeny after WHO susceptibility tests for *An gambiae *s.l. from Burkina Faso.

Goundry	Sample size	A	S	M	M/S	P Value
DDT Total	102	79	17	4	0	
DDT Survivors	22	5	77	18	0	0
DDT Dead	80	100	0	0	0	0
						
Permethrin Total	101	49	30	19	2	
Permethrin Survivors	24	29	50	21	0	0.007
Permethrin Dead	77	67	12	17	4	0.01

**Kuinima**						

DDT Total	104	22	68	10	0	
DDT Survivors	72	2	90	8	0	0
DDT Dead	32	66	19	15	0	0
						
Permethrin Total	105	28	61	10	1	
Permethrin Survivors	85	22	68	10	0	0.552
Permethrin Dead	23	48	39	9	4	0.008

**Koupela**						

DDT Total	105	71	15	14	0	
DDT Survivors	25	16	52	32	0	0
DDT Dead	80	88	4	8	0	0.007
						
Permethrin Total	104	60	14	26	0	
Permethrin Survivors	27	41	44	15	0	0
Permethrin Dead	77	67	29	4	0	0

**Soumousso**						

DDT Total	102	20	65	13	2	
DDT Survivors	80	7	78	13	2	0.059
DDT Dead	22	68	18	14	0	0
						
Permethrin Total	107	36	63	1	0	
Permethrin Survivors	58	5	95	0	0	0
Permethrin Dead	49	73	25	2	0	0

Only the results from round 2 (17-30^th ^October 2008) are presented. In all cases percentage mortality was ≤ 80%. Values are shown as percentage of total number of surviving or dead mosquitoes for each species. The total population represents the species distribution in the total population available for bioassay (see materials and methods for further details). The P value is the probability that the species distribution in the Survivors or Dead subset differs from the distribution in the total population.

All of the bendiocarb and fenitrothion survivors in Soumousso belonged to the S form of *An. gambiae*. A similar pattern was seen in Kuinima with all fenitrothion survivors and 46/47 bendiocarb survivors belonging to the S form (one M form *An. gambiae *survived bendiocarb exposure in Kuinima).

In Chad, permethrin and/or deltamethrin resistance was detected in all four sites but only Donia, the cotton growing area, showed resistance to DDT (Figure 3). All populations were fully susceptible to bendiocarb and fenitrothion. Previous insecticide bioassay data from collections made in August and September 2006, reported resistance to permethrin and deltamethrin in Bongor, the vector control site on the Cameroon border used in the current study [24]. Comparing the results from 2006 with those of round two in 2008 indicates an increase in the frequency of permethrin resistant individuals with mortality with the diagnostic dose decreasing from 59.8% mortality to 43% and, for deltamethrin, from 72.5% to 31% mortality over the two-year period.

*Anopheles arabiensis *was the only member of the *An. gambiae *complex identified in N'djamena and Mandelia and the predominant species in Bongor (Figures 3). In Donia, in the south of the country, *An. gambiae *M and S forms, and *An. arabiensis *were present in large numbers. All species/forms showed resistance to pyrethroids at this site.

In Sudan, as in the other countries, large heterogeneities were observed in mortality between the different sentinel sites. *Anopheles arabiensis *is the only member of the *An. gambiae *complex present in Gezira state (Figure 4 and [18,25]). Here, the highest numbers of bioassay survivors were observed in the urban agricultural site of Wad Medani, which is found within the Gezira irrigation scheme (Figure 4). Permethrin and DDT resistance was also observed in Roofa, which was selected as the low insecticide exposure site. In contrast, the cotton growing region of El Managil showed only 'suspected resistance' according to WHO definitions [13]. Permethrin and DDT resistance had been monitored in three of the four sites in Sudan in 2005/2006 [25]. There has been little decrease in mortality over the past three years in El Managil and only a small decrease in Roofa. In Wad Medani, mortality after DDT exposure was 89.9% in 2005/2006 and 95.1% after permethrin exposure. It is difficult to directly compare these results with the current study as a different age group of mosquitoes were used (1-3 days in [25] versus 3-5 days in the current study) and because the results from the current study show very strong seasonal variations, discussed below, that were not accounted for in the earlier study. Nevertheless, it appears as if the frequency of DDT and permethrin resistant individuals has increased in the past three years in Wad Medani. Mosquitoes from all four sites were fully susceptible to bendiocarb and fenitrothion.

## Discussion

Using WHO definitions of resistance [13], eight of the 12 populations in this study were resistant to DDT in at least one round of monitoring and a further three had suspected resistance. For the pyrethroids, all sentinel sites showed suspected or confirmed resistance to both permethrin and deltamethrin in at least one round of monitoring. Resistance to the acetylcholinesterase inhibitors, bendiocarb and fenitrothion, was more restricted with the four populations analyzed from Chad and the four from Sudan all remaining susceptible to these insecticide classes, although resistance is present in two sites in the southwest of Burkina Faso.

In the study design, sentinel sites with different levels of insecticide exposure were selected. The resources available did not permit a robust analysis of the effects of different selection pressures on the evolution of resistance but did enable a wider range of resistance phenotypes to be captured in each country. Surprisingly high levels of heterogeneities in resistance were observed even across relatively small distances. For example Wad Medani is less than 50 km southeast of El Magagil and yet, while the *An. arabiensis *population at the latter site showed only low or 'suspected resistance' to DDT and pyrethroids, the mortality rate to this insecticide was drastically reduced in Wad Medani.

Agricultural use of insecticides has been suggested to be one of the major drivers of insecticide resistance in malaria vectors [26,27]. Certainly the expansion of urban agriculture, which is often associated with intensive and under regulated insecticide use, is having a pronounced effect on the ecology and resistance levels in malaria vectors in several countries [28]. There are also many well-documented examples of IRS and LLINs directly selecting for insecticide resistance [8,29,30]. No IRS programmes are currently active in Burkina Faso or Chad and in these study sites the primary malaria vector control intervention is the use of LLINs. The distribution of these is very patchy within our study sites. Only Koupela in Burkina Faso and Bongor in Chad have been targeted for free net distribution to at risk populations but bed nets are used by some households in the other sites and the frequency of insecticide-treated nets in each site has not been documented or compared. In Sudan, larviciding with temephos is being used throughout Gezira State but only the Wad ElHadad site is targeted for IRS (with bendiocarb). LLINs can be found in all sites but, during the time of the study, coverage rates were highest in Wad ElHadad.

Pronounced differences were frequently observed in the mortality levels during the malaria transmission season. In general, higher numbers of bioassay survivors were observed towards the end of the malaria transmission season in Chad and Burkina Faso but this trend was reversed for DDT in two of the sites in these countries, Donia in Chad and Kuinima in Burkina Faso. Furthermore in Wad Medani, located in the Gezira irrigation scheme, lower survivorship was observed during the second collection season, which occurred in January 2009. The heterogeneity in resistance observed within short periods of times (typically four months elapsed between the beginning of the first monitoring round and the end of the second) may simply reflect stochastic variation and results from subsequent years will be needed to determine whether the trends observed in year one of the study are reproduced. In this study, bioassays were performed on adults raised from field caught larvae, in line with WHO recommendations [13]. Collecting immatures rather than raising progeny from blood fed females collected in houses increases the genetic heterogeneity of the samples and avoids selection biases that may be caused by collection of adults inside insecticide treated houses. A limitation of this approach is the lack of control over rearing conditions that would be achieved by using F1 progeny raised under standard conditions in the insectary. Hence the choice of using larval collections may have introduced additional 'noise' to the bioassay data. Nevertheless, there are several biological explanations that may account for this apparent seasonal heterogeneity.

Firstly, the results could reflect a change in species composition. *Anopheles gambiae *is a species complex composed of at least seven morphologically identical species, of which *An gambiae s.s *and *An arabiensis *are the most important in terms of malaria transmission. *Anopheles gambiae s.s *can be further divided into at least two different molecular 'forms'. Molecular analysis has found that the frequency of insecticide resistance alleles can vary dramatically within sympatric populations of *An. gambiae *s.l [31]. Therefore, the species and molecular form distribution was determined in both collection rounds in all sites. This was performed using a randomly selected subset of 40 mosquitoes from the control bioassay (Figure 2). However, it is important to note that, when the species distribution in this control subset was compared to the subset used for the insecticide bioassays in Burkina Faso, significant differences were observed in 3 of the 8 tests (data not shown). Further work in identifying the most appropriate sampling strategy to capture the species variation in a given study site is clearly needed.

Nevertheless, even when taking the above concerns into consideration, no clear trends in species distribution were observed that could explain the seasonal variation in insecticide susceptibility. For example, in Kuinima, Burkina Faso, the proportion of *An. gambiae *relative to *An. arabiensis *increased between collection rounds one and two. This was accompanied by an increase in the number of mosquitoes surviving pyrethroid exposure. However, in Soumousso, where the proportion of *An. gambiae *decreased between collection rounds, the frequency of pyrethroid resistant individuals in the population increased.

Many of the populations showed a significant difference in the species distribution of the survivors relative to the general population with the *An. gambiae *M or S form generally showing higher numbers of bioassay survivors than *An. arabiensis*. Care should be taken in interpreting these results due to sample size limitations imposed by the small number of survivors in some sites. However this analysis does demonstrate that resistance is not restricted to just one member of the *An. gambiae *complex and, while proportionally higher numbers of *An. gambiae s.s*. are generally observed in survivors, *An. arabiensis *is also resistant to pyrethroids.

In some sites, the change in observed susceptibility to insecticides between the two seasons may be related to exposure to insecticides in agriculture. The first round of mosquito collections in Wad Medani, Sudan, occurred in October/November 2008. During this time, crops in the Gezira irrigation scheme are regularly sprayed with insecticides. The second collection round occurred during the winter period, when crop production is lower and intensive insecticide spraying is reduced. The increased numbers of pyrethroid and DDT susceptible *An. arabiensis *individuals collected from breeding sites in this region in the winter months may possibly reflect this reduction in exposure to insecticides used in agriculture. A previous study in the cotton growing region of Pitoa, Northern Cameroon, observed a decrease in mortality between insecticide bioassays performed in July, at the start of the cotton spraying season, and those performed in late August/early September, mid way through the cotton crop and a period of intense insecticide application [26]. This observation was not repeated in the neighbouring cotton growing region of Donia, Chad, in this study. However, the increase in numbers of bioassay survivors in Soumousso seen in October versus August mirrors the decrease in mortality to pyrethroids reported during the rains in the cotton growing region of Burkina Faso in 1999/2000 [27].

A third explanation for the seasonal variation in mortality to pyrethroids in particular, is the cause for gravest concern. The results from Burkina Faso may be indicative of a very rapid selection for pyrethroid resistance in *An. gambiae s.s*. in West Africa. Although this network has only been in operation for one year, additional data from the region paint a worrying picture. The frequency of the *kdr *allele, which encodes an altered version of the voltage gated sodium channel and confers resistance to pyrethroids, is increasing at a rapid rate in the region [22]. Metabolic resistance mechanisms have also been described and, although tools are not yet available to track the frequency of these alleles as they spread through the population, laboratory analysis has identified several cytochrome P450 genes that are conferring pyrethroid resistance in multiple populations of *An. gambiae s.s *[32,33]. Similarly comparison of the results from the first year of this study with previously published reports from Chad and Sudan [24,25] also indicate rapid increases in the frequency of pyrethroid resistant mosquitoes. Clearly resistance monitoring, using both bioassays and molecular tools, needs to be incorporated as part of the monitoring and evaluation component of any existing or planned insecticide based malaria control activity. Selection of insecticides for IRS must consider the existing levels of insecticide resistance in the local vector populations and the alternative sources of selection pressure imposed on the local vector population.

With some notable exceptions [10,34] studies on the impact of insecticide resistance on malaria control activities are severely lacking, and indeed some studies have failed to observe any detrimental effect of resistance on mosquito control in experimental huts [35] but it would be naïve not to anticipate an eventual reduction in the efficacy of vector control interventions as a result of resistance. Indeed, a recent study in Benin, comparing the efficacy of LLINs and IRS in areas with largely susceptible or pyrethroid resistant populations of *An. gambiae s.l*., provided a stark reminder of how dependent malaria control in Africa is on a pyrethroid susceptible malaria vector population [36]. The current study shows that this trait cannot be taken for granted.

## Competing interests

The authors declare that they have no competing interests.

## Authors' contributions

HR is the PI of the WHO/TDR network. She conceived and designed the study and drafted the manuscript. HA, participated in the study design, led the field work in Sudan and carried out laboratory analysis. AB and WMG contributed to the field and laboratory work in Burkina Faso and the analysis of the data. CKH participated in the study design, led the field work in Chad and contributed towards the analysis. EYK contributed to the field work in Chad and carried out laboratory analysis. NFS participated in the study design, led the field work in Burkina Faso and contributed towards the analysis. FS participated in the study design, provided a critical review of the manuscript and participated in the data analysis for Chad. MC participated in the study design, provided a critical review of the manuscript and participated in the data analysis for Sudan. All authors read and approved the final manuscript.
